# Melatonin Mitigates Water Deficit Stress in *Cenchrus alopecuroides* (L.) Thunb through Up-Regulating Gene Expression Related to the Photosynthetic Rate, Flavonoid Synthesis, and the Assimilatory Sulfate Reduction Pathway

**DOI:** 10.3390/plants13050716

**Published:** 2024-03-03

**Authors:** Li Jiang, Minqiang Yun, Yinxi Ma, Tongbao Qu

**Affiliations:** College of Forestry and Grassland, Jilin Agricultural University, Changchun 130118, China; jiangli@jlau.edu.cn (L.J.); michardyi@jlau.edu.cn (M.Y.); 18391507030@163.com (Y.M.)

**Keywords:** melatonin, water deficit, photosynthetic, flavonoids, sulfur metabolism

## Abstract

Melatonin can improve plant adaptability to water deficit stress by regulating the biosynthesis of flavonoids and improving the reactive oxygen species-scavenging enzyme system. However, it remains unclear whether melatonin mitigates the effects and causes of water deficit stress in *Cenchrus alopecuroides*. We conducted a PEG-simulated water stress pot experiment to determine whether and how exogenous melatonin alleviates water deficit in *C. alopecuroides*. The experiment was divided into four treatments: (1) normal watering (Control), (2) 40% PEG-6000 treatment (D), (3) 100 μmol·L^−1^ melatonin treatment (MT), and (4) both melatonin and PEG-6000 treatment (DMT). The results showed that melatonin can alleviate water deficit in *C. alopecuroides* by effectively inhibiting plant chlorophyll degradation and MDA accumulation while increasing antioxidant enzyme activities and photosynthetic rates under water deficit stress. The transcriptome results indicated that melatonin regulates the expression of genes with the biosynthesis pathway of flavonoids (by increasing the expression of PAL, 4CL, HCT, and CHS), photosynthesis-antenna proteins (by increasing the expression of LHC), and sulfur metabolism (the expression of PAPSS and CysC is up-regulated in the assimilatory sulfate reduction pathway), while up-regulating the transcription factors (AP2/ERF-ERF-, C2H2-, WRKY-, Tify-, bHLH-, NAC-, and MYB-related). These findings revealed the possible causes by which melatonin mitigates water deficit stress in *C. alopecuroides*, which provided novel insights into the role of melatonin in water deficit stress.

## 1. Introduction

Global warming-induced water scarcity is the primary abiotic stress that restricts plant growth, development, and productivity [1]. A water deficit can cause changes in plant cell metabolism, resulting in the excessive accumulation of reactive oxygen species and lipid peroxidation, inhibiting plant photosynthesis and cell division, and ultimately leading to growth retardation and stagnation [2,3]. Developing effective solutions to mitigate the effects of water deficit on plants is critical [4]. Plants themselves have various mechanisms to combat stress by activating defense genes, encoding proteins involved in defense pathways, and activating antioxidant defense systems [5,6]. However, during a severe or prolonged water deficit, the defense system of plants can be affected, such as the ability to produce and activate antioxidant enzymes, which are then unable to cope with the resulting oxidative stress [7]. Priming using active small-molecule substances serves as an emerging approach to improve plant tolerance to abiotic stresses, such as melatonin [8].

Melatonin (chemical name: N-acetyl-5-methoxytryptamine, MT, or Mel) is a versatile small molecule compound belonging to the indole heterocyclic group [9]. Numerous studies have demonstrated that melatonin is involved in various physiological processes, including alleviating the inhibition of photosynthesis, promoting seed germination, regulating plant growth and root morphology [10,11,12,13,14], and regulating primary and secondary metabolism [15,16]. Under water deficit conditions, exogenous melatonin increased the expression of important genes such as superoxide dismutase (SOD), peroxidase (POD), and phenylalanine ammonia-lyase (PAL) in hickory (*Carya cathayensis*) [16]. Melatonin treatment improved photosynthetic efficiency by enhancing chlorophyll metabolism and increasing the expression of photosynthesis-antenna proteins [17]. As demonstrated in another study, the expression of genes involved in flavonoid biosynthesis such as phenylalanine ammonia-lyase (PAL), 4-coumarate-CoA ligase (4CL), shikimate-O-hydroxycinnamoyltransferase (HCT), and chalcone synthase (CHS) was up-regulated due to melatonin [18]. Melatonin also increased the accumulation of apigenin, lignans, and quercetin while activating stress-responsive transcription factors (TFs) such as NAC ERF, bHLH, and MYB, thereby reducing toxicity to maize roots [18,19]. Although melatonin has been shown to alleviate water deficit stress in a variety of plants, no studies have been conducted using melatonin to alleviate water deficit stress in *Cenchrus alopecuroides* (L.) Thunb. [syn. *Pennisetum alopecuroides* (L.) Spreng, Poaceae], and the mechanisms or causes underlying this process remain poorly understood.

*C. alopecuroides* is a forage and landscaping grass that is widely cultivated and is an affordable option with high value in garden applications due to its ease of planting and management [20,21,22]. However, *C. alopecuroides* growth is increasingly threatened by climate change and extreme water deficits as it intensifies, and it can be damaged by persistent water deficits, causing root/crown ratios to increase and aboveground biomass to decrease, ultimately resulting in lower yield [20,23]. To investigate the regulatory capacity of melatonin on water deficit in *C. alopecuroides*, we formulated the following hypotheses based on previous studies: (1) melatonin is able to alleviate water deficit effects on phenotypes, antioxidant enzymes, and photosynthesis in *C. alopecuroides* and (2) melatonin is able to modulate genes related to flavonoid biosynthesis and photosynthesis in *C. alopecuroides* during water deficit stress. To achieve the above, this study investigated the effects of melatonin on the growth, physiology, and molecules of *C. alopecuroides* during water deficit stress through physiology and transcriptomics. The present study’s results can clarify the possible ways in which melatonin enhances water deficit tolerance in *C. alopecuroides* and provide a theoretical basis for the application of melatonin in forage grass.

## 2. Results and Discussion

### 2.1. Plant Responses to Water Stress Induced by Melatonin

**Figure 1:**
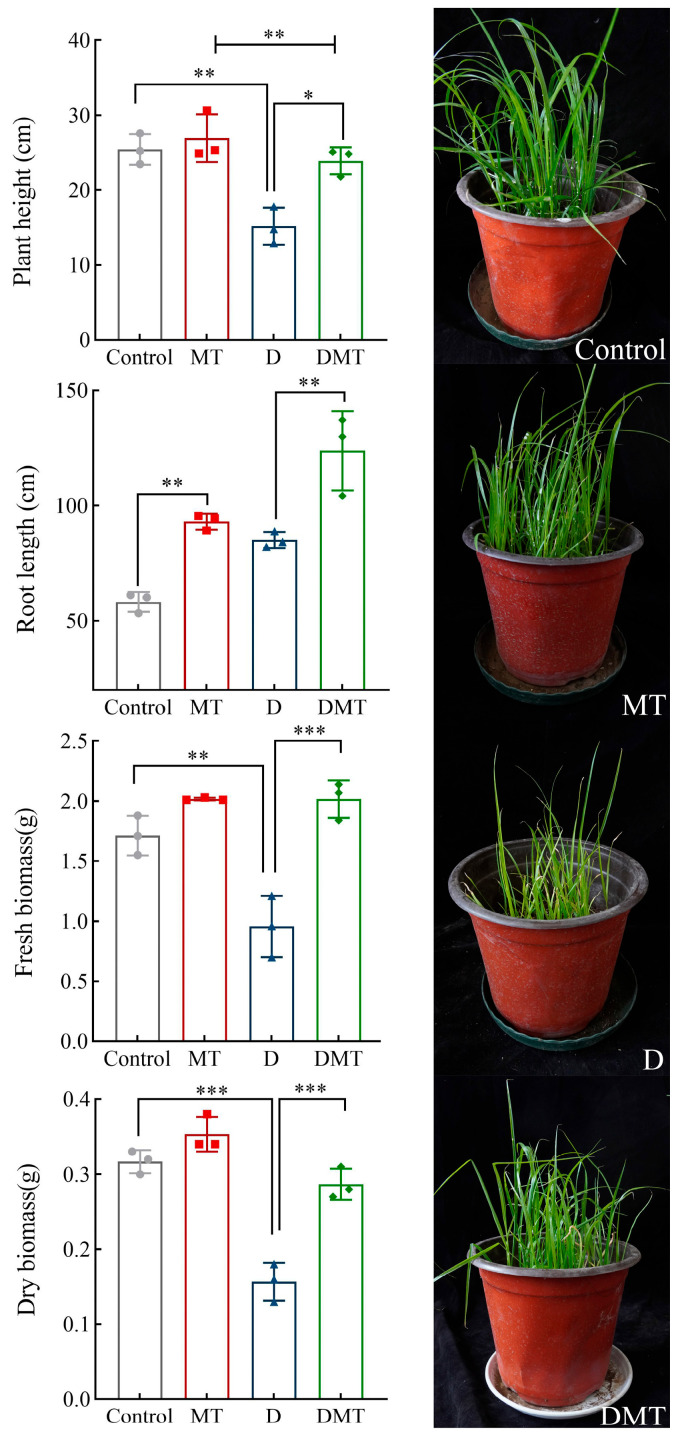
Effect of melatonin pretreatment on morphological indicators under water deficit stress. Note: * significantly correlated at the *p* < 0.05 level, ** significantly correlated at the *p* < 0.01 level, and *** very significantly correlated at the *p* < 0.001 level.

Water deficit stress caused plant growth retardation expressed in phenotypic traits, and the application of melatonin restored growth (Figure 1). Compared with the Control, the plant height, fresh biomass, and dry biomass decreased by 40.05%, 44.42%, and 50.40%, respectively, while the root length increased by 46.02% under water deficit stress (*p* < 0.01) (Figure 1). Compared with the water deficit group, significantly increased plant height, root length, fresh biomass, and dry biomass were observed in the DMT treatment, which increased by 57.58%, 45.49%, 110.89%, and 82.98% respectively (*p* < 0.01) (Figure 1). The present experiments and others have shown that melatonin improves plant height and biomass and increases root length by maintaining cell expansion and water balance, restoring palisade tissues, rationally distributing biomass, and expanding the range of root water uptake during water deficit stress [24,25,26,27]. This supports our hypothesis that melatonin can alleviate the impact of water deficits on phenotypic traits in *C. alopecuroides*. However, further investigation is required to determine the specific regulatory mechanisms. 

**Figure 2:**
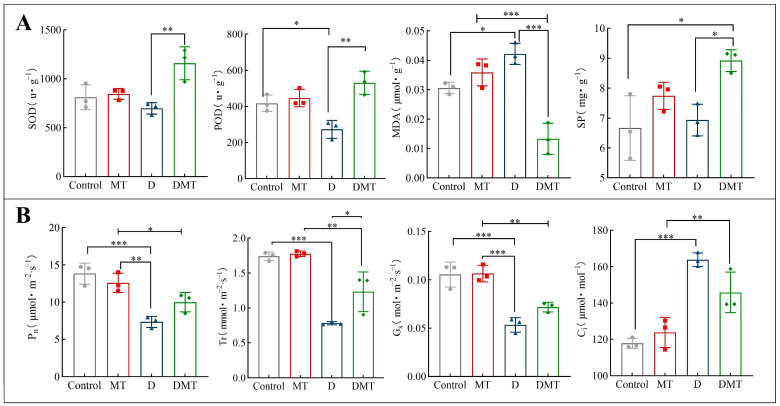
(**A**) Effects of melatonin pretreatment on relevant physiological indices under water deficit stress. (**B**) Effect of melatonin on gas exchange parameters under water stress. Note: * significantly correlated at the *p* < 0.05 level, ** significantly correlated at the *p* < 0.01 level, *** very significantly correlated at the *p* < 0.001 level.

The results showed that melatonin helps to maintain reactive oxygen species (ROS) homeostasis in plant cells, reduce membrane lipid peroxidation, and mitigate protein degradation. Compared with the Control, SOD and POD decreased by 14.02% and 34.53%, while MDA and SP increased by 37.91% and 3.97%, respectively, under water deficit stress (Figure 2A). Compared with group D, SOD, POD, and SP were significantly increased by 66.12%, 94.36%, and 28.71% and MDA was significantly decreased by 68.45% under the DMT treatment (*p* < 0.05) (Figure 2A). The experimental results and previous studies have shown that melatonin can improve the water retention capacity of a plant during water deficit stress by enhancing the antioxidant enzymes activity (SOD and POD) to scavenge free radicals, protect cell walls and lipid membranes from peroxidation, reduce protein degradation, and promote plant metabolism [11,28,29,30,31,32]. Melatonin regulates antioxidant enzymes through transcriptional expression of encoding key genes [33]. We also confirmed this argument in follow-up experiments. These findings also argue our hypothesis. However, in order to prove that melatonin can enhance plant water retention, it is necessary to conduct further studies on melatonin’s effect on gas exchange parameters.

### 2.2. Melatonin Improves Gas Exchange Parameters under Water Stress

The results showed that melatonin treatment significantly increased the photosynthetic rate, transpiration rate, and stomatal conductance of *C. alopecuroides* plants under water deficit stress. Compared with the Control group, the concentrations of Pn, Gs, and Tr were all significantly reduced by 46.93%, 49.43%, and 55.13% and Ci was increased by 39.06%, respectively, under water deficit stress (*p* < 0.001) (Figure 2B). Compared with the D group, the melatonin plus PEG-6000 treatment (DMT) significantly increased the Pn, Gs, and Tr concentrations by 36.04%, 34.48%, and 57.67% and Ci was decreased by 10.99%, respectively (*p* < 0.05) (Figure 2B). A negative correlation between Pn and Ci was found in the experiment, which may be caused by the increase in mesophyll conductance (g_m_) and mesophyll photosynthetic activity. Plants that experience water deficit stress show an inhibition of photosynthesis, with the primary response being the closure of the stomata to reduce transpiration, which reduces the rate of photosynthesis [34]. These results combined with previous studies have shown that melatonin can regulate photosynthesis and transpiration rates to improve water use efficiency, reduce stomatal water loss, increase photosynthetic pigments to mitigate water deficit damage to chloroplasts, and enhance the plant’s ability to tolerate during water deficit stress [30,35,36]. In a follow-up study, we observed that melatonin-pretreated plants increased chlorophyll content and regulated the expression of related genes under water stress. This supports the idea that melatonin restores photosynthetic performance under water stress and proves our hypothesis. 

### 2.3. Melatonin Modulates Transcriptomic Responses to Water Stress

#### 2.3.1. Transcriptome Sequencing Data and Quality Assessment

The transcriptome sequencing of twelve samples of *C. alopecuroides* leaves resulted in the minimum value of filtered Clean Reads at 39.64 M and the maximum at 48.68 M. The processed data percentage from the original dataset exceeded 91.3%, representing an increase from the original dataset. The transcriptome sequencing results indicated high quality, with each sample producing an average of 6.09 gigabytes of data and Q30 levels exceeding 94.36%. Additionally, the GC content ranged from 53.74% to 55.2% across all samples, confirming their appropriateness for subsequent analysis.

#### 2.3.2. Analysis of the Number of Differentially Expressed Genes

**Figure 3:**
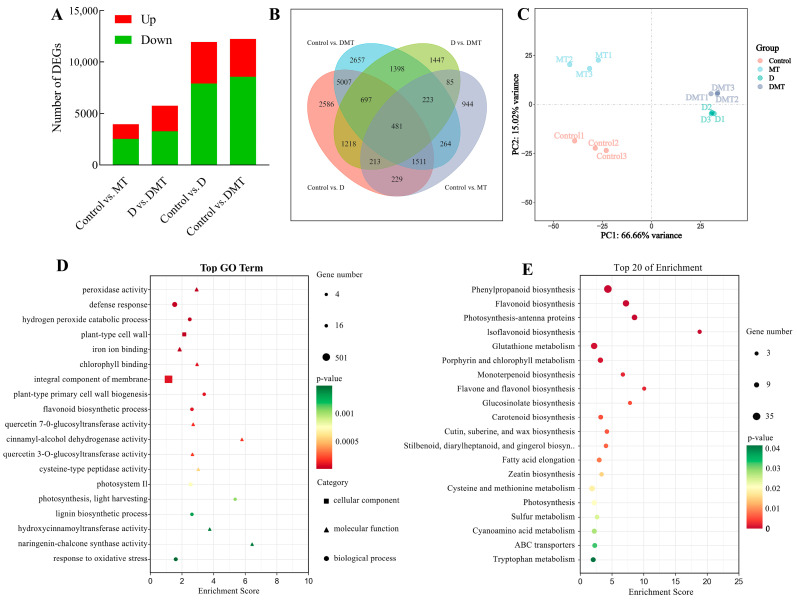
RNA-seq-based effects of exogenous melatonin on the genome expression profiles of *C. alopecuroides* seedlings under water deficit stress. (**A**) Number of DEGs for four comparisons. Red indicates up-regulated genes and green indicates down-regulated genes. (**B**) Venn plots of DEGs in the four comparisons. (**C**) Principal component analysis plots of transcriptome data for the four treatments. (**D**) Bubble plot of GO enrichment analysis of TOP20 in the D/DMT group. (**E**) Bubble plots of KEGG enrichment analysis for TOP20 in the up-regulated D/DMT group; larger bubbles indicate more enriched DEGs.

Based on sequencing data, differentially expressed genes (DEGs) were identified. The results showed that a total of 11,942 DEGs were screened in the Control/D group, including 4024 up-regulated and 7918 genes down-regulated. There was a total of 12,238 DEGs in the Control/DMT group, with 3664 genes up-regulated and 8574 genes down-regulated. There was a total of 5762 DEGs in the D/DMT group, with 2505 genes up-regulated and 3257 genes down-regulated (Figure 3A). These results indicated that melatonin-treated *C. alopecuroides* can regulate a higher number of genes in response to water deficit stress.

#### 2.3.3. GO and KEGG Enrichment Analyses

GO and KEGG enrichment analyses in the D/DMT group indicated the biological functions and metabolic pathways of melatonin-regulated DEGs under water stress. The GO enrichment analysis revealed that the set of genes that exhibit differential expression in the D/DMT comparison was significantly enriched into 189 terms (*p* < 0.05) (Figure 3D). Among these terms, 91 were for biological processes, 23 were for cellular components, and 75 were for molecular functions. The significantly enriched gene ontology terms included “peroxidase activity” (GO: 0004601), “defense response” (GO: 0006952), “flavonoid biosynthetic process” (GO: 0009813), “chlorophyll binding” (GO: 0016168), “integral component of membrane” (GO: 0016021), “photosystem II” (GO: 0009523), and “lignin biosynthetic process” (GO: 0009809), among others (Figure 3D). It was observed that most of these significantly enriched GO terms had up-regulated expression.

The KEGG enrichment analysis revealed that melatonin regulates secondary metabolic pathways that contribute to water deficit tolerance in *C. alopecuroides*. Our findings indicated that, under water deficit stress, exogenous melatonin up-regulates pathways such as “Phenylpropanoid biosynthesis” (ko00940), “Flavonoid biosynthesis” (ko00941), “Photosynthesis—antenna proteins” (ko00196), “Isoflavonoid biosynthesis” (ko00943), “Glutathione metabolism” (ko00480), “Cutin, suberine, and wax biosynthesis” (ko00073), and “Sulfur metabolism” (ko00920) pathways (Figure 3E). Melatonin mainly regulates genes for phenylpropanoid synthesis and photosynthetic reactions at the transcriptome level during water deficit stress. Previous and present studies have shown that melatonin can help plants realize morphological adaptation and precise regulation of metabolic pathways under adverse conditions [29,37]. Phenylpropane metabolism, one of the important secondary metabolic pathways in plants, produces a variety of metabolites such as flavonoids, lignans, cinnamic acid amides, etc., which play an important role in regulating the adaptive growth of plants [38,39]. We observed that the precursor enzymes of these substances were regulated. In addition, photosynthesis is highly sensitive to environmental stress, resulting in the disruption of chloroplast structure, a reduction in the photosynthetic rate, a limitation of electron transport, and damage to pigmentation complexes [40,41]. Our experiments detected that melatonin regulates DEGs in the photosynthesis-antenna proteins and porphyrin and chlorophyll metabolic pathways.

### 2.4. Melatonin Regulates Gene Transcription Levels in Secondary Metabolic Pathways

#### 2.4.1. Melatonin Regulates the Phenylpropanoid and Flavonoid Synthesis Pathways

**Figure 4:**
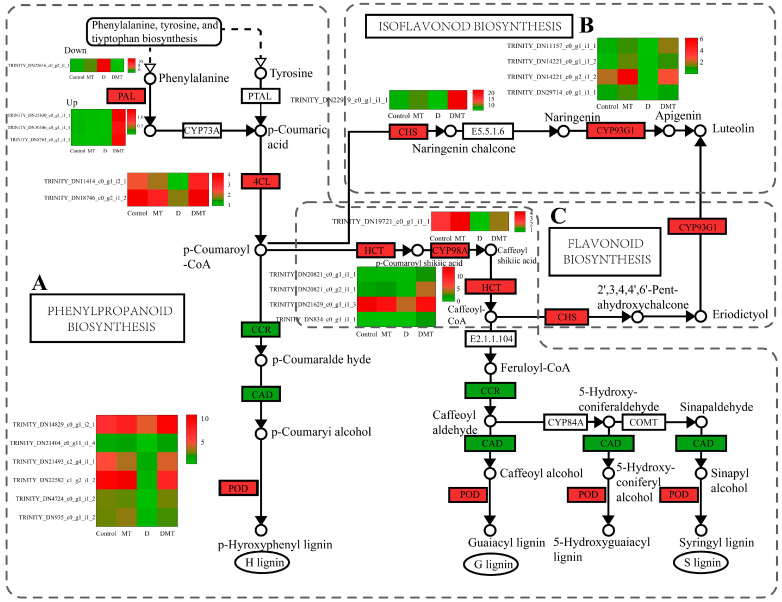
Melatonin regulates oxidoreductase production, lignin synthesis and signaling pathways, and gene expression quantity (FPKM) in the D/DMT group. (**A**) Phenylpropanoid biosynthesis pathways. (**B**) Isoflavonoid biosynthesis pathways. (**C**) Flavonoid biosynthesis pathways. Red represents the up-regulation of this genome and green represents the down-regulation of this genome.

Melatonin regulated the precursor enzymes for the synthesis of flavonoids including lignin, apigenin, and luteolin in the phenylpropanoid biosynthesis, flavonoid biosynthesis, and isoflavonoid biosynthesis pathways (Figure 4). In the D/DMT comparison, water stress inhibited the expression of the majority of the genes in these pathways. However, the application of exogenous melatonin significantly up-regulated PAL, 4CL, HCT, 5-O-(4-coumaroyl)-D-quinate 3′-monooxygenase (CYP98A), and peroxidase (POD) expression (*p* < 0.05), while the DEGs of cinnamoyl-CoA reductase (CCR) and cinnamyl alcohol dehydrogenase (CAD) were significantly downregulated. The expression of the DEGs of chalcone synthase (CHS) and flavone synthase II (CYP93G1) was significantly up-regulated (*p* < 0.05) (Figure 4). PAL, 4CL, HCT, and CYP98A could promote the production of lignin from cinnamic acid, coumaric acid, and caffeic acid through molecular interactions and enzymatic reactions, while CHS and CYP93G1 could catalyze the generation of compounds such as apigenin, naringenin, and luteolin from 4-coumaroyl-CoA (Figure 4). Melatonin up-regulated the expression of these genes, possibly allowing lignin, apigenin, and luteolin to accumulate as a result. Lignin and apigenin have been reported to help plants attenuate cellular damage caused by unfavorable environmental factors [42,43]. Luteolin and its derivatives exhibit exceptional properties in scavenging ROS and protecting cells during water deficit stress [44]. This experiment combined with previous studies suggested that melatonin may enhance the antioxidant capacity of plants by regulating DEGs in the phenylpropanoid and flavonoid synthesis pathways [39,43,45,46,47]. However, these results need to be further confirmed by rigorous field trials.

#### 2.4.2. Melatonin Regulates Photosynthesis-Antenna Proteins

**Figure 5:**
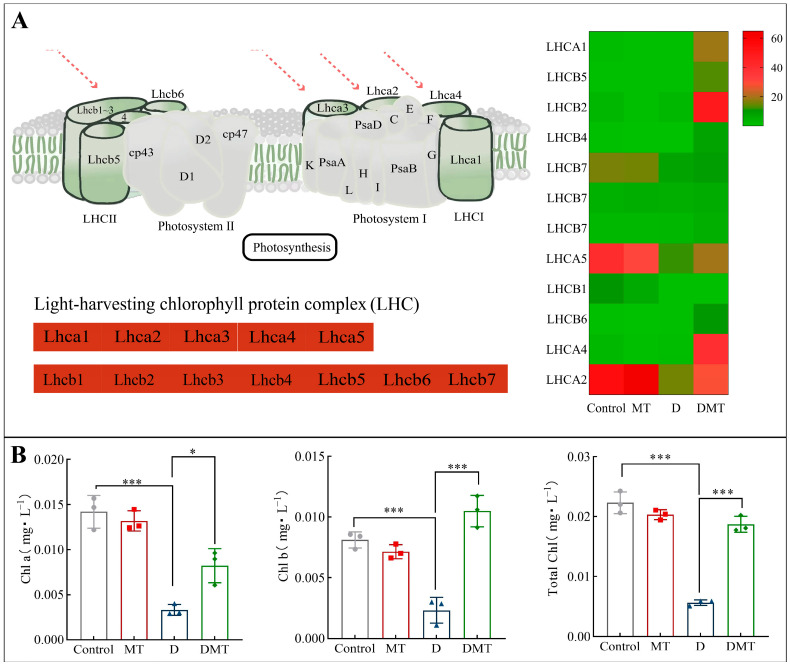
(**A**) Melatonin regulates the photosynthesis-antenna protein pathway and expression quantity (FPKM) in the D/DMT group. Red represents the up-regulation of this genome. (**B**) Changes in chlorophyll a, chlorophyll b, and total chlorophyll content of *C. alopecuroides* in the four treatments. Note: * Significantly correlated at the *p* < 0.05 level and *** very significantly correlated at the *p* < 0.001 level.

Compared with the water deficit group, melatonin significantly up-regulated the expression of Light-trapping chlorophyll a/b binding (LHC) protein and increased plant chlorophyll content (Figure 5). In total, melatonin up-regulated 15 DEGs in the LHCA, with a differential fold change ranging from 21.42 to 101.2. The 18 DEGs in the LHCB were also up-regulated, and their differential fold change ranged from 4.03 to 37.43. Meanwhile, compared with the PEG-6000 stress (D) treatment, the melatonin plus PEG-6000 treatment (DMT) significantly increased total chlorophyll, chlorophyll a, and chlorophyll b by 66.56%, 148.14%, and 352.39%, respectively (*p* < 0.001) (Figure 5). The level of chlorophyll content reflects to some extent the strength of photosynthesis in a plant. Plant photosynthesis is the process of converting light energy into free energy for life activities through a series of reactions, realizing the acquisition of the sun’s energy as well as its storage [48,49]. Light-harvesting chlorophyll-binding (LHC) proteins enhance light absorption and stimulate energy transfer. In addition, LHCA1 to LHCA5 as well as LHCB1 to LHCB7, members of the LHC gene family [49], play a crucial role in photosynthesis. LHC proteins promote photochemical quenching and optimize photosynthesis by directing light energy to the reaction centers of PSI and II through compounds formed by the reaction [50]. When analyzed with other studies, the results showed that melatonin can enhance plant photosynthesis and improve plant resistance to water stress by regulating LHC in photosynthesis-antenna proteins to improve light energy uptake and transfer, increasing photosynthetic pigments and mitigating water deficit damage to chloroplasts [30,50]. This corresponds to the previously described improvement in gas exchange parameters and is consistent with our hypothesis. 

#### 2.4.3. Melatonin Regulates the Assimilatory Sulfate Reduction (ASR) Pathway of Sulfur Metabolism

**Figure 6:**
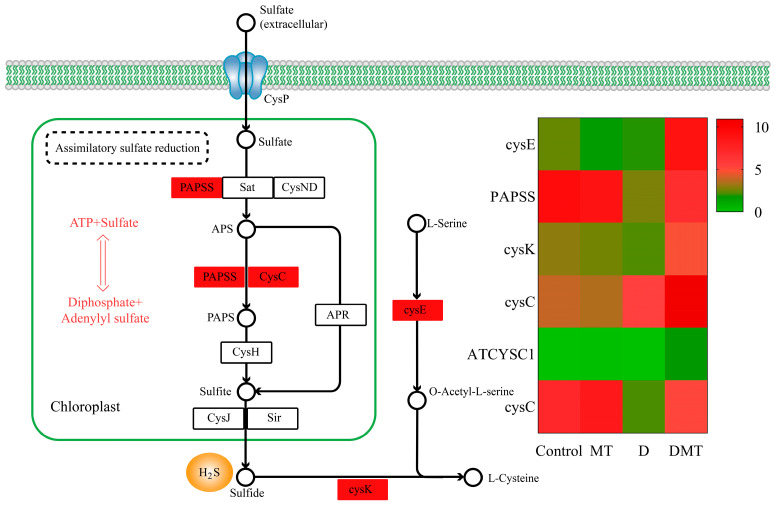
Melatonin regulates the assimilatory sulfate reduction (ASR) pathway of sulfur metabolism and gene expression quantity (FPKM) in the D/DMT group. Red represents the up-regulation of this genome.

During our investigation into the causes by which melatonin may regulate water stress, we discovered pathways that go beyond the initial hypothesis. It was observed that melatonin regulated the assimilatory sulfate reduction (ASR) pathway of sulfur metabolism. We discovered six significantly up-regulated DEGs in the sulfur metabolic pathway. The ASR pathway was the primary concentration of six differentially expressed genes, with water stress leading to decreased expression of 3′-phosphoadenosine 5′-phosphosulfate synthase (PAPSS) and adenylylsulfate kinase (CysC). However, compared with water deficit, exogenous melatonin up-regulated the gene expression of PAPSS and CysC, promoting sulfide fixation and production (*p* < 0.05) (Figure 6). PAPSS and CysC are the key enzymes for endogenous hydrogen sulfide (H_2_S) production in the ASR pathway [51]. In different environments, ASR is an active pathway for sulfate reduction [52]. Sulfate was activated in the ASR pathway through PAPSS, resulting in the production of adenosine monophosphate (AMP), followed by the formation of 3′-phosphoadenosine-5′-phosphosulfate sulfite (PAPS) [52]. Subsequently, sulfide was generated through a series of enzymatic steps involving various enzymes like CysC [52]. Sulfur is crucial for the development of all living organisms [53]. Plants absorb and convert inorganic sulfur present in the environment, especially SO_4_^2−^ from the soil and sulfur dioxide gas from the air, into organic sulfur [53]. Moreover, sulfide is an emerging signaling molecule in the regulation of water deficit resistance in plants [54]. Studies have shown that exogenous melatonin increased endogenous H_2_S production in *Arabidopsis thaliana* under water stress and that H_2_S could respond to abiotic stress by closing stomata, regulating the transcriptional levels of the relevant genes, influencing post-translational modifications of proteins, and interacting with other signaling molecules [54,55]. Our study provided novel insights that melatonin may regulate the assimilatory sulfate reduction pathway to promote endogenous sulfide production and then regulate stomatal closure to enhance water deficit tolerance in *C. alopecuroides*.

### 2.5. RT-qPCR Validation of Transcriptome Data

The accuracy and reproducibility of transcriptomics data were verified by assessing the transcript abundance of five selected DEGs through RT-qPCR analysis. The expression trends in the RNA-seq data were consistent with the qPCR gene expression patterns (Figure S1), indicating that our RNA-seq data had high reliability, reproducibility, and accuracy.

### 2.6. C. alopecuroides Transcription Factors Are Affected by Exogenous Melatonin 

Exogenous melatonin activates transcription factors (tf) under PEG water deficit stress. In this study, we identified 346, 394, and 442 differentially expressed transcription factors from 48 families in Control/D, Control/MT, and D/DMT respectively (Figure S2). Melatonin increased differential genes in AP2/ERF-ERF-, C2H2-, WRKY-, Tify-, bHLH-, NAC-, and MYB-related families under water deficit stress. Among them, AP2/ERF-ERF-, WRKY-, and MYB-related genes were the most numerous, at 17, 17, and 11, respectively. TFs play important roles in regulating genes related to plant development and external stimulus responses. For instance, AP2/ERF, WRKY, bHLH, MYB, NAC, and bZIP can sense stress signals, activate stress-responsive genes, and regulate signaling pathways, contributing to crop adaptation to abiotic stress [8,56]. Exogenous melatonin has been reported to activate the AP2-ERF, NAC, MYB, bHLH, and WRKY families in the maize root system in response to water deficit stress [18]. This is consistent with our findings. This suggests that exogenous melatonin has the potential to alleviate water deficit stress in *C. alopecuroides* by activating AP2/ERF-ERF-, C2H2-, WRKY-, Tify-, bHLH-, NAC-, and MYB-related families. However, the direct link between TFs and plant water deficit stress response needs further verification due to the relative complexity of the mechanisms of water deficit and MT-induced plant changes.

### 2.7. Correlations between Environmental Factors and Related Genes in the Growth of C. alopecuroides under Different Treatments

**Figure 7:**
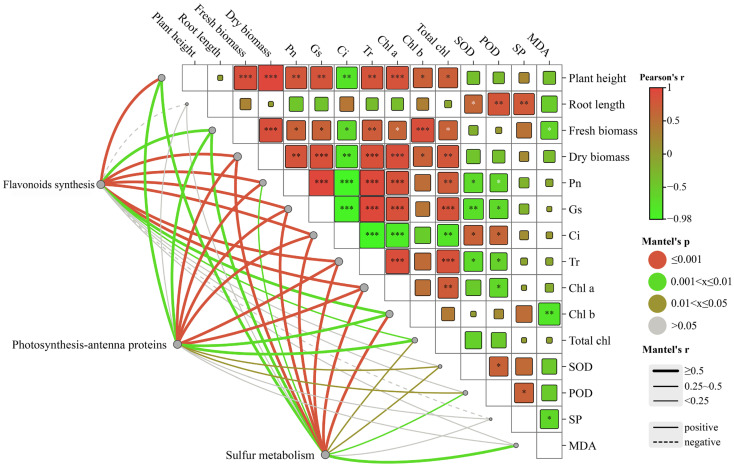
Mantel test correlation study between environmental factors and related genes in the growth of *C. alopecuroides*. Note: The lines represent the correlation between related genes and environmental factors, with thicker lines indicating stronger correlations. Solid lines: positive correlations. Dotted lines: negative correlations. *: *p* < 0.05, **: *p* < 0.01, and ***: *p* < 0.001.

The Mantel test analysis revealed a significant correlation between transcribed genes and photosynthetic genes (Figure 7). The results suggested that differentially expressed genes in the flavonoid synthesis pathways were significantly correlated (r < 0.9, *p* = 0.001) with plant height and dry weight. Additionally, significant correlations (r < 0.6, 0.001 < *p* < 0.005) were observed between the genes and gas exchange parameters and chlorophyll. Negative correlations were found between the genes and soluble proteins among the three growth indicators. Genes expressed differently in the photosynthesis-antenna protein pathway exhibited significant correlations with the net photosynthetic rate, stomatal conductance, intercellular CO_2_ concentration, and transpiration rate (r < 0.8, *p* = 0.001). Moreover, they showed correlations with SOD and POD (r < 0.5, *p* < 0.03). The genes that were differentially expressed in the sulfur metabolic pathway showed significant correlation with plant height, fresh weight, gas exchange parameters, and chlorophyll (r < 0.8, *p* < 0.005), as well as with SOD, POD, and MDA. The correlation between changes in environmental factors and the differentially expressed genes in *C. alopecuroides* under different treatments suggested that melatonin may confer water deficit resistance to plants by influencing photosynthetic rates through the regulation of transcriptional expression of plant secondary metabolites.

## 3. Materials and Methods

### 3.1. Plant Material and Growth Conditions

*C.alopecuroides* seeds were provided by a garden company, and the experiment was conducted in the Jilin Agricultural University Artificial Climate Chamber (125°24′58″ E, 43°48′37″ N). Intact and plump seeds were soaked in 1% NaClO for 15 min for surface sterilization. The seeds were washed five times with deionized water and subsequently soaked in deionized water for 24 h. A total of 100 soaked seeds were sown in plastic pots (16 cm in high × 18 cm in caliber) and a soil base of a garden soil/nutrient soil (2/1) mixture. The pots were placed in an artificial climate chamber with a temperature of 26 ± 2 °C, humidity of 50–70%, a light/dark cycle of 15/9 h, and a light intensity of 1800 lx and were watered once every 3 days to maintain soil moisture.

### 3.2. Experimental Designs

The optimal treatment of melatonin to alleviate PEG-simulated water deficit stress in *C. alopecuroides* was determined in a pre-experiment, that is, foliar spraying of 100 μmol·L^−1^ melatonin under 40% PEG-6000 stress. After 40 days of growth in pots, they were divided into four groups: (1) normal watering (Control), (2) PEG-6000 treatment (D), (3) melatonin treatment (MT), and (4) both melatonin and PEG-6000 treatment (DMT). Three biological replicates were performed for each group. Except for the normal watering and PEG-simulated water deficit groups, the leaves of the remaining potted plants were sprayed with 100 mL of 100 μmol∙L^−1^ melatonin solution. After 3 consecutive days of spraying, 100 mL of 40% PEG-6000 solution was applied to the potted plants requiring water deficit stress. After 7 days, morphology and related physiological indexes were determined. Some samples were frozen in liquid nitrogen and stored at −80 °C for transcriptome sequencing.

### 3.3. Growth of Morphological and Physiological Indicators

#### 3.3.1. Growth of Morphological Indicators

Plant height and root length were measured by straightedge and vernier caliper. Using scales (Lichen, Shanghai, China) with 1/10,000 accuracy, the fresh and dry biomasses were measured.

#### 3.3.2. Measurement of Physiological Indicators

The SOD activity was determined through the nitrogen blue tetrazolium method [57]. First, 1 g of fresh leaves was crushed in 5 mL phosphate buffer and centrifuged at 4 °C (10 min, 10,000 rpm). The color reaction was then carried out, and the supernatant was mixed with other solutions. Phosphate buffer, EDTA, methionine, NBT, riboflavin, and supernatant made up the reaction mixture. Finally, the absorbance was measured at 560 nm by using a UV spectrophotometer (Shanghai Jingke, Shanghai, China).

The POD activity was determined through the guaiacol color development method [58]. First, 1 g of fresh leaves was crushed in 10 mL phosphate buffer and centrifuged (15 min, 4000 rpm). Then, 1 mL of supernatant was added to 3 mL of the reaction mixture. The light absorption value at 470 nm was measured by using a spectrophotometer (Shanghai Jingke, Shanghai, China) and was recorded every minute. The reaction mixture consisted of potassium phosphate buffer, hydrogen peroxide, and guaiacol.

Malondialdehyde (MDA) is the end product of fatty acid degradation, an indicator of lipid peroxidation. We used the thiobarbituric acid reactive substances (TBARS) method to determine the content of MDA in plant leaves [59]. First, 1 g of fresh leaves was pulverized in 5 mL of 10% trichloroacetic acid (TCA) and centrifuged at 4000 rpm for 10 min. Subsequently, 2 mL of supernatant was collected to form a mixture with 2 mL of 0.5% thiobarbituric acid (TBA); the mixture was reacted in a boiling water bath for 15 min, cooled rapidly, and then centrifuged. The absorbance of the supernatant was measured at 532 and 600 nm.

The soluble protein (SP) content was measured using the Coomassie brilliant blue G-250 staining method [60]. First, 1 g of fresh leaves was crushed in 5 mL phosphoric acid buffer and centrifuged (10 min, 4000 rpm). Next, we added the supernatant to the Coomassie brilliant blue solution and performed colorimetry at 595 nm with the help of a spectrophotometer (Shanghai Jingke, China). Finally, we calculated the soluble protein content based on the standard curve.

The photosynthetic rate (Pn), stomatal conductance (Gs), inter-cellular CO_2_ concentration (Ci), and transpiration rate (Tr) of the main stem leaves and the inverted trifoliate leaves were measured with a portable photosynthesis system analyzer Li-6400 (Li-Cor, Lincoln, NE, USA). We used a closed-circuit air circuit with a built-in light source and a cylinder to control the CO_2_ concentration and set the CO_2_ concentration at 400 μmol·mol^−1^, the flow rate at 500 μmol·s^−1^, and the light intensity at 1200 μmol·m^−2^·s^−1^. The experiment was conducted at a controlled ambient temperature of 25 °C and relative humidity of 70%. Three plants were selected for each treatment and measured between 9:00 and 11:00 am on a sunny day. 

Chlorophyll levels were determined using the ethanol–acetone mixture immersion method [60]. A fresh sample weighing 1 g was cut and soaked in a 10 mL mixture of 95% ethanol and 80% acetone in a 1:1 ratio for storage in darkness. The concentrations of chlorophyll a and chlorophyll b were calculated by measuring light absorption values at wavelengths of 663 nm and 645 nm through the spectrophotometer (Shanghai Jingke, Shanghai, China).

All of the reagents mentioned above were purchased from Changchun Anmei Biotechnology Co. (Changchun, China).

### 3.4. RNA Sequencing Experimental Method

#### 3.4.1. RNA Isolation and Library Preparation

Using TRIzol reagent, RNA was extracted. RNA purity and quantity were evaluated by using a NanoDrop 2000 spectrophotometer (Thermo Scientific, Waltham, MA, USA), and RNA integrity was assessed by using an Agilent 2100 Bioanalyzer (Agilent Technologies, Santa Clara, CA, USA). Using VAHTS Universal V6 RNA-seq Library Prep (Illumina, San Diego, CA, USA), libraries were constructed. After passing QC, sequencing was performed by an Illumina Novaseq 6000 (Illumina, San Diego, CA, USA). In Shanghai, China, Shanghai Ouyi Biotechnology performed transcriptome sequencing and analysis.

#### 3.4.2. RNA Sequencing Analysis Process

Trimmomatic was used to process the raw fastq data. Clean reads were obtained by removing reads containing polyN and low quality. The assembly of clean reads into clusters of expressed sequence tags (contigs) was followed by de novo assembly into transcripts using Trinity (version: 2.4) with paired-ends. Based on similarity and length, the longest transcript was selected as the unigene for subsequent analyses.

The unigenes’ function was annotated by aligning them with the NCBI non-redundant (NR), Swiss-Prot, evolutionary genealogy of genes Non-supervised Orthologues (eggNOG) and Eukaryotic Complete Genomes (KOG) databases using diamond with a threshold of e < 1 × 10^−5^. The proteins with the highest hits to the unigenes were used to assign functional annotations. The unigenes were mapped to the Kyoto Encyclopedia of Genes and Genomes (KEGG) database for potential pathway annotation. Gene Ontology (GO) classification was performed by mapping Swiss-Prot and GO terms. Hierarchical cluster analysis of differentially expressed genes (DEGs) was conducted using R (v 3.2.0) to illustrate the expression pattern of unigenes in different groups and samples. GO and KEGG pathway enrichment analyses were performed using R based on the hypergeometric distribution.

### 3.5. Real-Time Fluorescence Quantitative PCR (RT-qPCR)

To evaluate the precision and reproducibility of sequencing data, we selected significant DEGs for RT-qPCR validation. The internal reference gene chosen for this assay was GAPDH. The assay was performed by Shanghai Ouyi Biotechnology Co. (Shanghai, China).

### 3.6. Statistical Analysis of Data

SPSS 26.0 was used for statistical analysis and GraphPad Prism for plotting. The effects of the four treatments on growth indices and relevant physiological indices were analyzed by one-way ANOVA. The LSD method was used to compare the significance between treatments (*p* < 0.05). The relationship between growth environment factors and relevant genes was analyzed by the Mantel test.

## 4. Conclusions

**Figure 8:**
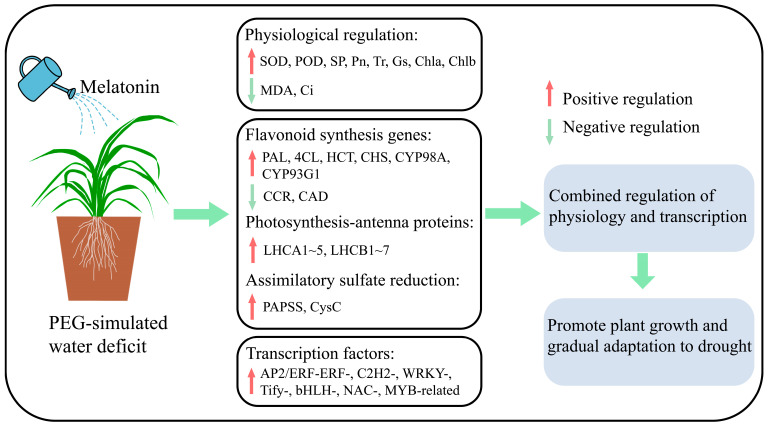
A simple mechanistic model for melatonin mitigation of water deficit stress in *C. alopecuroides*.

In this study, the application of exogenous melatonin is found to modulate the growth, physiology, and gene expression of *C. alopecuroides* plants during water deficit stress conditions, ultimately increasing their water deficit tolerance. We propose a simple mechanism model (Figure 8). Melatonin is able to increase the activity of antioxidant enzymes, inhibit the accumulation of MDA, and improve gas exchange parameters. Furthermore, melatonin up-regulates the expression of PAL, 4CL, HCT, CHS, CYP98A, and CYP93G1 in flavonoid biosynthesis, LHC in photosynthesis-antenna proteins, and PAPSS and CysC in the assimilatory sulfate reduction pathway. The expression of AP2/ERF-ERF-, C2H2-, WRKY-, Tify-, bHLH-, NAC-, and MYB-related families and a series of downstream transcription factors was induced to improve water deficit stress. The study results offer valuable insights into the molecular mechanism of melatonin in mitigating water stress in pasture grasses. This will enhance the theoretical basis and open up new research directions for utilizing melatonin technology to improve water deficit resistance in pasture grasses in the future.

## Data Availability

Data are available on request.

## Supplementary Materials

The following supporting information can be downloaded at: https://www.mdpi.com/article/10.3390/plants13050716/s1, Figure S1: (A) FPKM trends in DEGs analyzed by RNA-seq. (B) Relative expression trends in DEGs verified by RT-qPCR. Figure S2: Heat map of transcription factor families in three comparisons: D/DMT, Control/D, and Control/DMT.
