# Supplementary material for: Melatonin Mitigates Water Deficit Stress in Cenchrus alopecuroides (L.) Thunb through Up-Regulating Gene Expression Related to the Photosynthetic Rate, Flavonoid Synthesis, and the Assimilatory Sulfate Reduction Pathway
Source: Plants (Basel). 2024 Mar 3;13(5):716. doi: 10.3390/plants13050716 (PMC10934972; doi:10.3390/plants13050716)
Supplement: Supplementary file 1 [file plants-13-00716-s001.zip › plants-2860443-supplementary.pdf]

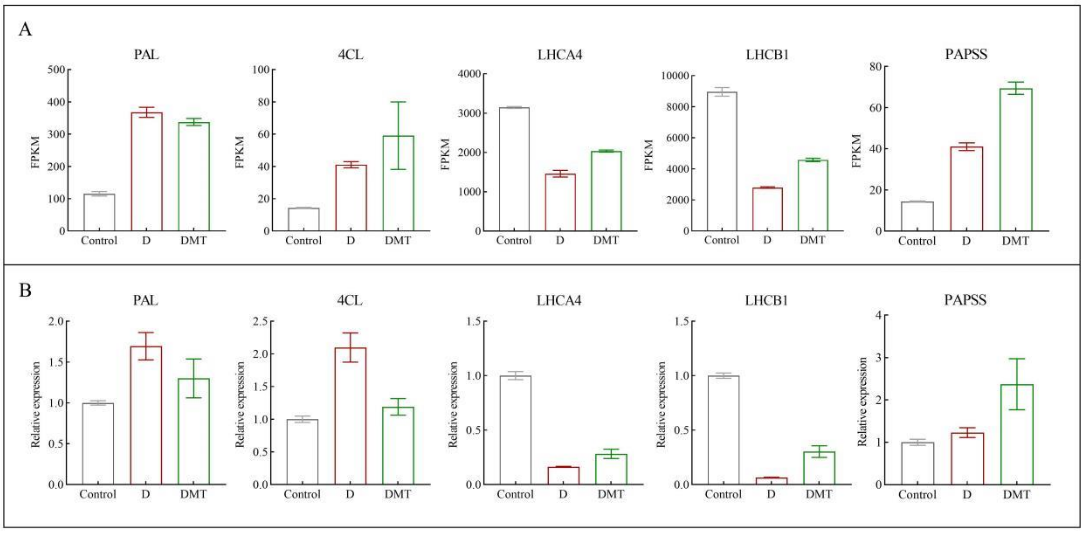

Figure. S1 (A) FPKM trends in DEGs analyzed by RNA-seq. (B) Relative expression trends in DEGs verified by RT-qPCR.

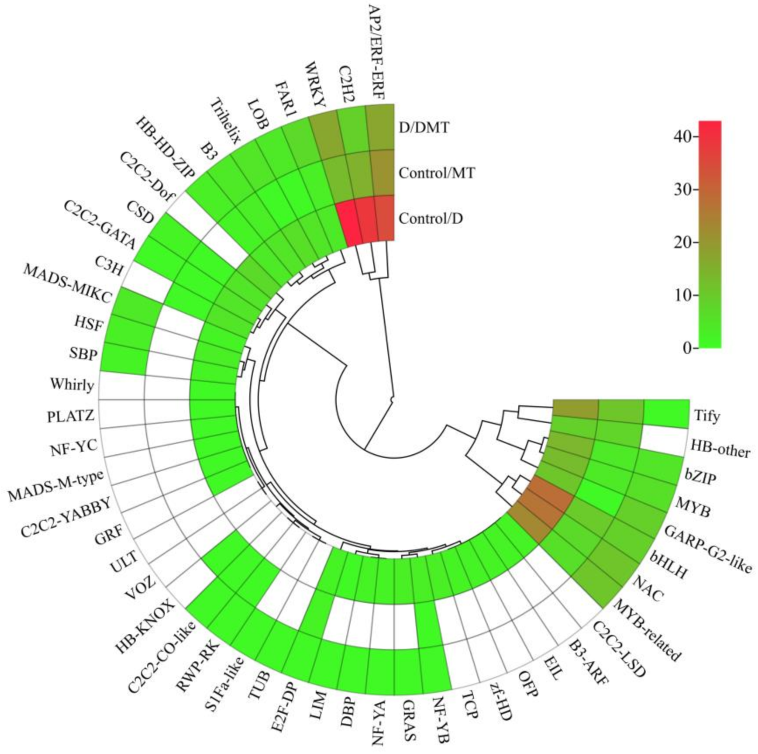

Figure. S2 Heat map of transcription factor families in three comparisons: D/DMT, Control/D and Control/DMT.
